# Highly Pathogenic Avian Influenza Virus H5N1 Infects Alveolar Macrophages without Virus Production or Excessive TNF-Alpha Induction

**DOI:** 10.1371/journal.ppat.1002099

**Published:** 2011-06-23

**Authors:** Debby van Riel, Lonneke M. E. Leijten, Menno van der Eerden, Henk C. Hoogsteden, Leonie A. Boven, Bart N. Lambrecht, Albert D. M. E. Osterhaus, Thijs Kuiken

**Affiliations:** 1 Department of Virology, Erasmus MC, Rotterdam, The Netherlands; 2 Department of Pulmonary Medicine, Erasmus MC, Rotterdam, The Netherlands; 3 Department of Immunology, Erasmus MC, Rotterdam, The Netherlands; 4 Department of Clinical Chemistry, Meander MC, Amersfoort, The Netherlands; 5 Laboratory of Immunoregulation and Mucosal Immunology, University of Ghent, Ghent, Belgium; University of Wisconsin-Madison, United States of America

## Abstract

Highly pathogenic avian influenza virus (HPAIV) of the subtype H5N1 causes severe, often fatal pneumonia in humans. The pathogenesis of HPAIV H5N1 infection is not completely understood, although the alveolar macrophage (AM) is thought to play an important role. HPAIV H5N1 infection of macrophages cultured from monocytes leads to high percentages of infection accompanied by virus production and an excessive pro-inflammatory immune response. However, macrophages cultured from monocytes are different from AM, both in phenotype and in response to seasonal influenza virus infection. Consequently, it remains unclear whether the results of studies with macrophages cultured from monocytes are valid for AM. Therefore we infected AM and for comparison macrophages cultured from monocytes with seasonal H3N2 virus, HPAIV H5N1 or pandemic H1N1 virus, and determined the percentage of cells infected, virus production and induction of TNF-alpha, a pro-inflammatory cytokine. *In vitro* HPAIV H5N1 infection of AM compared to that of macrophages cultured from monocytes resulted in a lower percentage of infected cells (up to 25% vs up to 84%), lower virus production and lower TNF-alpha induction. *In vitro* infection of AM with H3N2 or H1N1 virus resulted in even lower percentages of infected cells (up to 7%) than with HPAIV H5N1, while virus production and TNF-alpha induction were comparable. In conclusion, this study reveals that macrophages cultured from monocytes are not a good model to study the interaction between AM and these influenza virus strains. Furthermore, the interaction between HPAIV H5N1 and AM could contribute to the pathogenicity of this virus in humans, due to the relative high percentage of infected cells rather than virus production or an excessive TNF-alpha induction.

## Author Summary

Alveolar macrophages (AM), which reside in the alveolar lumen, usually dampen down the host immune response to incoming pathogens. However, they are thought to increase inflammation during highly pathogenic avian influenza virus (HPAIV) H5N1 infections, which cause severe and often fatal disease in humans. This is based on experiments with human macrophages cultured from monocytes rather than with human AM. Here we show that human AM, collected via broncho-alveolar lavage from healthy volunteers, can become infected with HPAIV H5N1. However, this results in neither induction of the pro-inflammatory cytokine TNF-alpha nor virus production. Therefore, AM are most likely not responsible for the excessive cytokine response or high viral load during human HPAIV H5N1 infections as assumed previously. These data significantly changes our insight into the pathogenesis of HPAIV H5N1 pneumonia in humans, indicating that other cells than AM must be responsible for the excessive pro-inflammatory cytokine profile observed during HPAIV H5N1 infections.

## Introduction

Seasonal, pandemic and zoonotic influenza A virus infections cause substantial morbidity and mortality in humans. Seasonal influenza virus infections in humans are usually mild, causing pneumonia in a minority of infected individuals. Pandemic influenza virus infections vary in their disease outcome. The 1918 Spanish flu caused pneumonia more often than the recent pandemic H1N1 (pH1N1) virus. Zoonotic influenza virus infections in humans vary from self-limiting conjunctivitis to severe, often fatal pneumonia. The currently ongoing outbreak of highly pathogenic avian influenza virus (HPAIV) of the subtype H5N1 in poultry is sporadically transmitted to humans, in which it causes severe pneumonia with a case-fatality rate of about 60% [1], [2], [3].

The differences in pathogenesis of different influenza virus infections in humans, and thereby their disease outcome are only partly understood. Early events after infection of the human alveoli—the site of pneumonia—contribute to protection against or the development of pneumonia. Previously we have shown that different influenza viruses attach to different cell types in the human alveoli. Seasonal influenza viruses and pH1N1 virus attach predominantly to type I pneumocytes [4], [5], whereas HPAIV H5N1 attaches predominantly to type II pneumocytes and alveolar macrophages (AM) [6]. This fits with the detection of influenza A virus antigens in type II pneumocytes in lung tissues from HPAIV H5N1 fatal infections [7], [8], [2]. Furthermore, in experimental HPAIV H5N1 infections of *ex vivo* lung cultures, influenza virus antigen was detected in type II pneumocytes and alveolar macrophages [9], [10]. Therefore, the response of alveolar epithelial cells and AM early after HPAIV H5N1 infection is likely important in the development of disease. In the present study we focus on the AM, an important cell of the innate immune system in the human alveolus.

Lung macrophages can be distinguished in alveolar, pleural, interstitial and intravascular macrophages, but the AM is the most important cell of the innate immune system in the human alveoli [11]. The phenotype of the AM is induced by the micro-environment of the human alveoli, which includes interactions with epithelial cells, the presence of surfactant proteins A (SP-A) and D (SP-D) and the presence granulocyte-macrophage colony stimulating factor (GM-CSF) [12], [13]. This phenotype resembles that of alternatively activated macrophages or deactivated macrophages, which were first described in 1992 [14], [15], [16], [17]. Precursors of AM are monocytes that enter the lung [11], after which they mature into the AM phenotype [18].

The AM has a protective role in the lung to prevent loss of function. Therefore, immune responses need to be balanced to protect the lungs from invading pathogens without excessive inflammation [18]. AM phagocytose virus particles and apoptotic cells by which they protect the lung from influenza induced damage [19], [20]. Depletion of AM leads therefore to enhanced virus replication and more severe disease during influenza virus infection [21], [22].

The role of the AM in the pathogenesis of HPAIV H5N1-induced pneumonia has been studied by *in vitro* infections of macrophages cultured from monocytes in the presence of human serum (MM). In MM, HPAIV H5N1 infects up to 100% of the cells, which results in a productive infection. Infection of MM with HPAIV H5N1 also results in an excessive immune response, marked by the high induction of the pro-inflammatory cytokine TNF-alpha, which is not observed after infection with seasonal H1N1 or H3N2 virus [23], [24], [25]. However, AM and MM are phenotypically different cells, where AM have a alternatively activated phenotype, whereas MM have a classical activated phenotype [15]. Furthermore, MM respond differently to influenza virus infections than AM as was shown by Ettensohn and Roberts [26]. In that study, MM produced significantly higher levels of interferon than AM. Another study revealed that only up to 20% of AM were infected with seasonal influenza viruses, and did not result in a productive infection [27]. Therefore, it is unclear whether MM are suitable substitutes for studying the interaction between influenza virus and AM.

To address this question, we compared the effect of seasonal H3N2 virus, pH1N1 virus or HPAIV H5N1 infection both on AM from broncho-alveolar lavages (BAL) of healthy volunteers, and on MM. In addition, we determined the percentage of AM infected in experimentally infected *ex vivo* cultured lung biopsies. Also we examined whether monocytes cultured in the presence of GM-CSF (GM-MM)—which is abundantly present in the alveolar lining fluid—instead of human serum would develop a phenotype more similar to that of the AM [28].

## Results

### Percentage of cells infected in ex vivo lung biopsies

**Figure 1:**
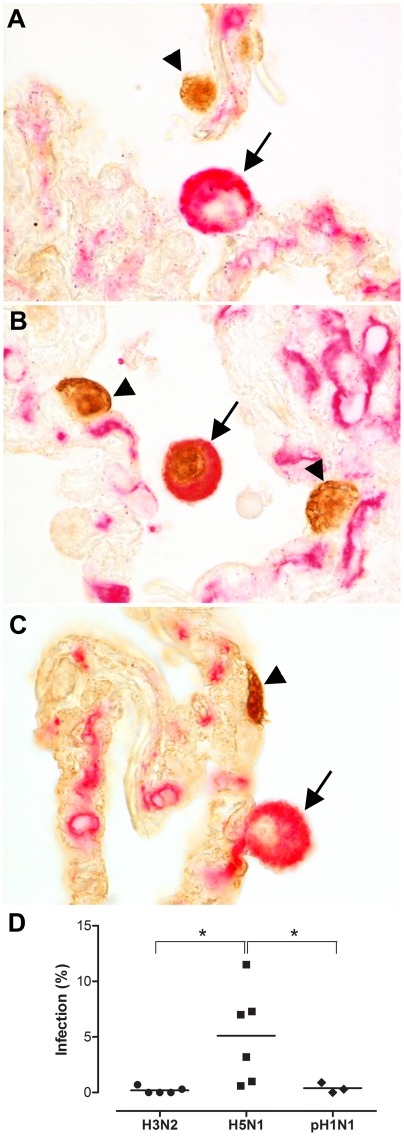
Alveolar macrophages in *ex vivo* lung cultures. Double staining for influenza A virus nucleoprotein (brown nuclear staining) and a macrophage marker (red/pink, cytoplasmic staining) 24 hours after infection with seasonal H3N2 virus (a), HPAIV H5N1 virus (b) or pandemic H1N1 virus (c). The arrowhead indicates influenza virus staining in alveolar epithelial cells and the arrow indicates an alveolar macrophage (AM). Seasonal H3N2 virus infection results in infection of alveolar epithelial cells (arrowhead) but not of AM (arrow). HPAIV H5N1 infection results in the infection of alveolar epithelial cells (arrowhead) and infection of AM (arrow). Pandemic H1N1 virus infection results in infection of alveolar epithelial cells (arrowhead) but not of AM (arrow). Percentages of AM infected after 24 hpi with seasonal H3N2 virus, HPAIV H5N1 or pH1N1 virus infection (d). Mean values are represented by horizontal lines. * indicates a statistical (p<0.05) difference.

All three viruses, seasonal H3N2 virus, pH1N1 virus and HPAIV H5N1, infected AM in experimentally infected *ex vivo* lung cultures, although percentages varied largely. In the double-staining, influenza virus antigen was present in the nucleus, indicative for virus replication, whereas HAM65, a macrophage marker, was present in the cytoplasm (Figure 1a–c). HPAIV H5N1 infected significantly more AM than seasonal H3N2 or pH1N1 viruses in *ex vivo* cultured lung biopsies (Figure 1d).

### Phenotype of macrophages

**Figure 2:**
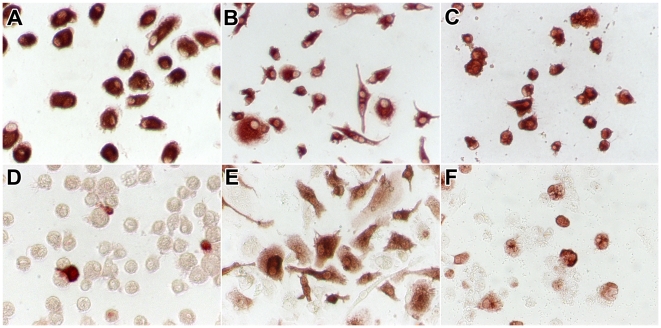
CD68 and influenza A virus antigen expression in alveolar macrophages and macrophages cultured from monocytes. Macrophages included were alveolar macrophages (a, d), macrophages cultured from monocytes in the presence of human serum (b, e), and macrophages cultured from monocytes in the presence of GM-CSF (c, f). In uninfected cells, staining for CD68 (a–c) confirms that these cells were of the monocyte/macrophage lineage. Twenty-four hours after infection with HPAIV H5N1, staining for influenza A virus nucleoprotein (d–f) confirms that these cells were infected by influenza virus.

AM had a round shape (Figure 2a). The shape of macrophages cultured from monocytes depended on the culture medium in which they differentiated. Most MM (cultured in the presence of human serum) were spindle-shaped with a few round cells (Figure 2b); in contrast, most GM-MM (cultured in the presence of GM-CSF) were round with a few spindle-shaped cells (Figure 2c). All cells were confirmed to be of the macrophage/monocyte lineage by CD68 staining (Figure 2a–c).

### Percentage of AM, MM and GM-MM infected

**Figure 3:**
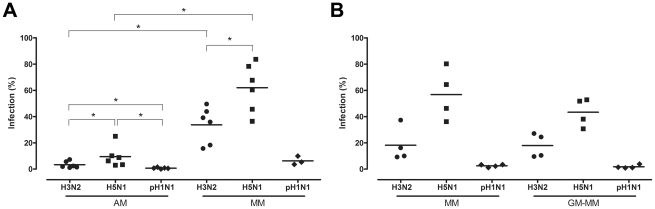
Percentage of alveolar macrophages and monocyte-derived macrophages infected with H3N2, H5N1 or H1N1 virus. Percentage of infected alveolar macrophages (AM) and monocyte-derived macrophages cultured in the presence of human serum (MM), from the same donor (a). Percentages of infected MM or macrophages cultured in the presence of GM-CSF (GM-MM), cultured from the same blood bank donors (b). Mean values are represented by horizontal lines. * indicates a statistical (p<.05) difference.

Influenza A antigen was visible as nuclear and cytoplasmic red staining (Figure 2d–f). Significant less AM than MM from the same donor were infected with either seasonal H3N2 virus or HPAIV H5N1 (Figure 3a). The percentage of AM infected by influenza virus declined significantly in the following order: HPAIV H5N1 > seasonal H3N2 virus > pH1N1 virus (Figure 3a). The same pattern was observed for MM and GM-MM (Figure 3b) cultured from the blood bank donors. For each of the three viruses tested, the percentage of cells infected did not differ significantly between MM cultured from the BAL- and blood bank donors, or between the MM and GM-MM from the blood bank donors (Figure 3a–b).

### Virus production in AM, MM and GM-MM

**Figure 4:**
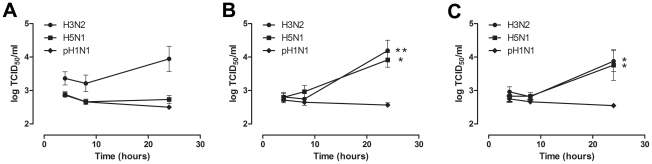
Virus production in alveolar macrophages and monocyte-derived macrophages after H3N2, H5N1 or H1N1 virus infection. Virus production of alveolar macrophages (AM) (a), macrophages cultured in the presence of human serum (MM) (b), and macrophages cultured in the presence of GM-CSF (GM-MM) (c), after infection with seasonal H3N2 virus, HPAIV H5N1 or pandemic H1N1 virus. Geometric mean titers were calculated from independent experiments; error bars indicate standard deviation. * indicates a statistical (p<0.05) difference. ** indicates a statistical (p<0.01) difference.

There was no significant influenza virus production in AM regardless of the virus used (Figure 4a). In contrast, there was significant production of seasonal H3N2 virus and HPAIV H5N1 in both MM and GM-MM (Figure 4b and 4c). There was no significant pH1N1 virus production in any of the cell types used.

### Cytokine production in AM and MM after virus inoculation or LPS exposure

**Figure 5:**
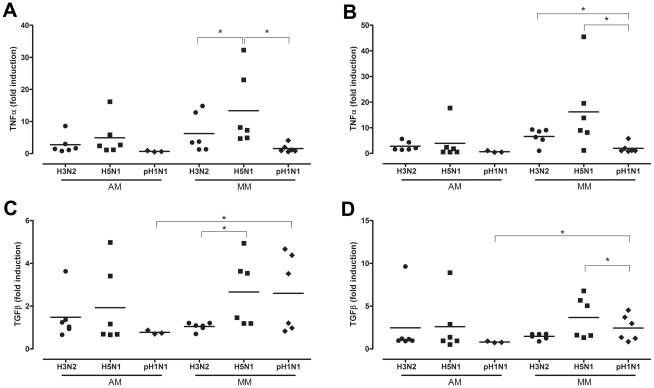
Cytokine mRNA levels after H3N2, H5N1 or H1N1 virus infection. TNF-alpha mRNA levels expressed in fold-induction over non-infected cells after seasonal H3N2 virus, HPAIV H5N1 or pandemic H1N1 virus infection in alveolar macrophages (AM) and macrophages cultured in the presence of human serum (MM) at 8 hpi (a) and 24 hpi (b). The same for TGF-beta mRNA levels at 8 hpi (c) and 24 hpi (d). Mean values are represented by horizontal lines. * indicates a statistical (p<0.05) difference.

HPAIV H5N1 infection of AM did not induce significant more TNF-alpha mRNA levels than seasonal H3N2 virus or pH1N1 virus 8 and 24 hpi (Figure 5a and 5b). In contrast, and as found previously [23], this was the case for HPAIV H5N1 virus infection of MM. There was a trend (p = 0.055) for HPAIV H5N1 infection to induce more TNF-alpha in MM than AM, both at 8 and 24 hpi (Figure 5a and 5b). The results for TGF-beta were similar for those of TNF-alpha, although overall the levels of induction were lower: HPAIV H5N1 infection did not induce significantly more TGF-beta than seasonal H3N2 virus or pH1N1 virus in AM, but did in MM (Figure 5c and 5d). Interestingly, pH1N1 virus infection of MM induced significantly more TGF-beta than that of AM, both at 8 hpi and 24 hpi. Since pH1N1 does not show measurable replication in MM, this induction might be a response of phagocytosis of virus proteins only.

**Figure 6:**
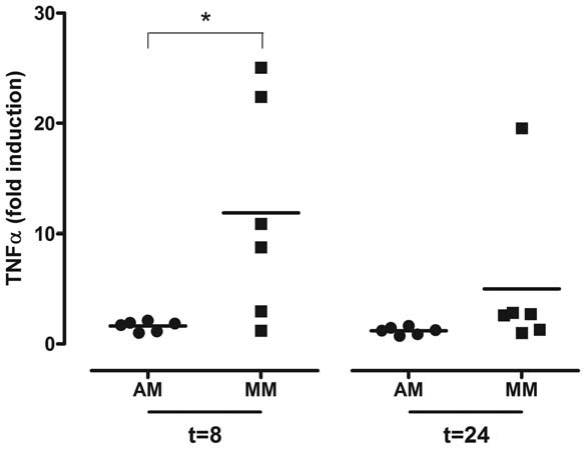
TNF-alpha mRNA levels in alveolar macrophages and monocyte-derived macrophages after LPS exposure. TNF-alpha mRNA levels expressed in fold-induction over non-infected cells after LPS exposure of alveolar macrophages (AM) or monocyte-derived macrophages (MM) at 8 hpi and 24 hpi. Mean values are represented by horizontal lines. * indicates a statistical (p<0.05) difference.

After exposure to LPS, there was significantly more TNF-alpha induced in MM than AM 8 hpi. Although this trend was still present 24 hpi, this difference was no longer significant at 24 hpi (p = 0.06) (Figure 6). This inhibition of TNF-alpha induction in AM is indicative for the alternative activation phenotype, in contrast to the classical activation phenotype of MM.

## Discussion

This is the first time that the role of AM during HPAIV H5N1 virus infection has been studied. This study shows that AM are more susceptible to HPAIV H5N1 infection than to seasonal H3N2 or pH1N1 virus infection but, that this infection results neither in virus production nor in an excessive immune response.

Macrophages cultured from monocytes do not respond the same way as AM to two out of three influenza viruses included in this study: there are significant differences between MM and AM in the percentages of cells infected, cytokine response and virus production after infection with HPAIV H5N1 and seasonal H3N2 virus. Therefore, MM are not suitable to study the interaction between AM and these influenza virus strains.

HPAIV H5N1 infected more AM than seasonal H3N2 or pH1N1 viruses, in both the *ex vivo* lung cultures and in AM collected from BAL. This fits with the results of our virus attachment studies, in which HPAIV H5N1 attached more abundantly to AM than H3N2 virus [4] and pH1N1 [5]. Infection of an AM likely hampers its protective function and, since approximately one AM is found in each alveolus [11], this could lead to substantial loss of protection in the alveoli.

HPAIV H5N1 infection in AM did not result in significantly higher induction of TNF-alpha mRNA compared to seasonal H3N2 or pH1N1 virus. This is in contrast with the significantly higher TNF-alpha mRNA levels in MM after HPAIV H5N1 virus infection compared to seasonal H3N2 and pH1N1 virus infection observed in our and previous studies [23], [24], [25]. The observed difference in TNF-alpha induction can likely be explained by the activation phenotype of AM compared to that of MM. MM represent classical activated macrophages, while AM are alternative activated or deactivated macrophages. LPS is known to induce TNF-alpha production in classically activated macrophages but not in alternatively activated macrophages [29], [17], [16]. The inhibited TNF-alpha induction of AM in this study fits with their alternatively activated phenotype. This inhibition of TNF-alpha induction in alternatively activated macrophages is thought to be regulated by the p50 subunit of NF-κB [30].

TNF-alpha, and the cascade it induces, is known to attract neutrophils and other leukocytes in the lung and is thought to play an important role in the pathogenesis of acute respiratory distress syndrome and multiple organ dysfunction syndrome [31]. The lack of excessive TNF-alpha induction in AM after infection with the HPAIV H5N1 strain used in this study might indicate that, in contrast to previous conclusions based on studies with MM, the AM does not contribute to the excessive immune response after HPAIV H5N1 infections.

The lack of virus production in AM after infection with influenza viruses included in this study corresponds with a previous study in which no virus release in AM was detected after infection with a seasonal H1N1 or H3N2 virus [27]. The lack of significant virus production indicates that AM do not release high numbers of new virus particles and thereby do not contribute to a productive infection in the human alveolus.

During influenza virus pneumonia, there is an influx of a variety of leukocytes, including monocytes [1]. The monocytes that enter the alveoli during influenza virus pneumonia most likely respond differently than resident AM. We cannot exclude that these monocytes will respond in a similar way as MM, with high percentages of cells infected, a productive infection and the induction of TNF-alpha. Unfortunately, the interaction between monocytes and influenza virus has never been studied, so their role remains to be elucidated.

Interestingly, macrophages that were cultured in the presence of GM-CSF, resembled AM in shape, but differed in their response to influenza virus infection. In fact, the percentages of cells infected of GM-MM resembled that of the MM more closely. Although GM-CSF is abundantly present in the human alveolus and is thought to be responsible for the AM phenotype [32], [33], [28], addition of GM-CSF was not sufficient to transform the phenotype of monocytes to that of AM, with respect to their response to influenza virus infections. It is likely that other factors present in the alveolus are required for this transformation.

Overall, the pH1N1 virus used in this study did not infect high percentages of either AM or MM. Even compared to seasonal H3N2 virus, pH1N1 virus infected lower percentages of cells. Whether this is a unique feature of pH1N1 virus or a common feature of pandemic influenza viruses remains to be determined. Overall, this observation fits with the relatively mild disease caused by pH1N1 virus infection [34].

In conclusion, we have shown that the MM are unsuitable to study the interaction between AM and these influenza virus strains, which might be due to their difference in phenotype; alternatively activated versus classically activated. In contrast to MM, AM do not induce excessive TNF-alpha after HPAIV H5N1 infection. However, AM are more abundantly infected by HPAIV H5N1 than by seasonal H3N2 virus or pH1N1 virus. We speculate that this relatively high percentage of AM infection by HPAIV H5N1 may contribute to the unusual high pathogenicity of HPAIV H5N1 for the human lung.

## Materials and Methods

### Ethics statement

The collection of AM via a broncho-alveolar lavage was approved by the Dutch Medical Ethical Committee (METC Erasmus MC, Rotterdam, The Netherlands, MEC-2008-018). All study participants provided written informed consent for the collection of samples and subsequent analysis.

### Viruses

Viruses used for these experiments were seasonal H3N2 virus (A/Netherlands/213/03), a pandemic H1N1 virus (A/NL/602/09) and a HPAIV H5N1 (A /Vietnam/1194/04). All virus stocks were prepared on Madin-Darby Canine Kidney cells (MDCK).

### Percentage of AM infected in ex vivo lung cultures

Surgically removed lung tissues, without any histological evidence for respiratory disease, were used for *ex vivo* infections. Biopsies were made with a 3-mm-biopsy punch and cultured overnight in F12K (Gibco, Breda, The Netherlands) supplemented with 100 U/ml penicillin, 100 µg/ml streptomycin, 2 mM glutamine and 5% FCS. After washing, to remove FCS, lung biopsies were infected with 10^7^ tissue culture infectious dose (TCID)_50_/ml of seasonal H3N2 virus, pH1N1 virus or HPAIV H5N1 at room temperature on a rocker for 1 hour. Lung biopsies were washed and cultured in F12K supplemented with 100 U/ml penicillin, 100 µg/ml streptomycin and 2 mM glutamine at 37°C in 95% O_2_ and 5% CO_2_. After 24 hours, biopsies were collected in 10% neutral-buffered formalin, embedded in paraffin, and sectioned at 3 µm.

All lung biopsy sections were stained for pancytokeratin to determine the condition of the alveolar epithelium. Lung biopsy sections of which the epithelium in more than 50% of the alveoli was desquamated were excluded. Lung biopsy sections infected with seasonal H3N2 virus (n = 5) pH1N1 virus (n = 3), HPAIV H5N1 (n = 6), or uninfected lung biopsy sections were stained for influenza A virus nucleoprotein as described earlier [35]. To determine the percentage of AM infected, sections were double-stained for influenza A virus nucleoprotein and HAM65 (macrophage marker). Slides were incubated with an antibody against influenza A virus nucleoprotein (IgG2a, Clone Hb65, American Type Culture Collection, Wesel, Germany), followed by incubation with an antibody against HAM65 (IgM, DAKO, Glostrup, Denmark). Primary antibodies were detected with a mixture of peroxidase-labeled goat-anti-mouse IgG2a (Southern Biotech, Birmingham, AL, USA) and alkaline phosphatase-labeled goat-anti-mouse IgM (Southern Biotech). Peroxidase activity was revealed with 3,3′–diaminobenzidine-tetrachlorhydrate (DAB) (Sigma, St. Louis, MO, USA), resulting in a brown precipitate. Alkaline phosphatase was revealed with fast red TR/naphthol AS-MX (Sigma), resulting in a pink precipitate. All macrophages present in the alveolar lumina were counted as influenza A positive or -negative. The number of AM present in the lung biopsy sections varied between 36 and 223 per section. In total 684 AM were counted in H3N2-infected lung biopsy sections, 741 AM in HPAIV-H5N1-infected lung biopsy sections and 590 AM in pH1N1-infected lung biopsy sections.

### Isolation and culture of alveolar macrophages

Six non-smoking volunteers (age >18 years) free of any respiratory symptoms, with a normal lung function (FEV_1_ >85% predicted, Tiffeneau-index >0.7), and a normal thorax X-ray underwent a BAL. The BAL was performed according to standard clinical procedures. A total of 3 times 50 ml warm, sterile 0.9% NaCl was instilled and aspirated sequentially. The middle pulmonary lobe was selected for sampling in all cases.

From each donor, the BAL was filtered to remove debris and mucus. Subsequently, AM from each donor were independently plated in the required concentrations in serum-free macrophage medium (SFM, Gibco) supplemented with 100 U/ml penicillin, 100 µg/ml streptomycin and 2 mM glutamine. After 2 hours, non-adherent cells were removed. To confirm that cells isolated were macrophages, cells were stained for CD68 (KP-1, Dako) using the same protocol as for the influenza nucleoprotein staining described below.

### Culture of monocyte-derived macrophages from heparine blood

From each donor that underwent the BAL, peripheral blood mononuclear cells (PBMC) were isolated from heparinized blood using a Lymphoprep gradient (density 1.007±0.001 g/ml (Axis-shield PoC AS)). Subsequently, monocytes were purified using a Percoll gradient (density 1.063±0.002 g/ml (GE healthcare, London, UK)). Monocytes from each donor were cultured in suspension at a concentration of 1*10^6^ cells/ml in teflon flasks (Nalgene, Roskilde, Denmark) in RPMI-1640 (Lonza, Walkersville, MD, USA) supplemented with 100 U/ml penicillin, 100 µg/ml streptomycin, 2 mM glutamine and 5% human AB serum. After 7 days, monocyte-derived macrophages (MM) from each donor were harvested from the teflon flask and seeded independently in flat-bottom culture plates. After 2 hours, non-adherent cells were removed. Cells were stained for CD68 as described below.

### Culture of monocyte-derived macrophages from blood bank donors

Blood was diluted in PBS and centrifuged for 10 minutes at 220 *g*. The top layer, mainly consisting of thrombocytes, was discarded. After this extra procedure, PBMC and monocytes were subsequently isolated and purified as described above. For comparison, from each blood bank donor, half of the monocytes were cultured in teflon flasks as described above, and the other half was cultured in RPMI supplemented with 100 U/ml penicillin, 100 µg/ml streptomycin, 2 mM glutamine, 5% FCS and GM-CSF (25 ng/ml, Invitrogen, Paisley, UK). GM-CSF cultured MM (GM-MM) were harvested after 7 days. Cells were stained for CD68 as described below.

### Percentage of cells infected

AM (n = 6), MM (n = 10, 6 paired samples with AM and 4 from blood bank donors) and GM-MM (n = 4) were seeded in a concentration of 50 000 cells per wells. MM obtained from peripheral blood of the AM donors were compared directly to AM from the same donor in below infection experiments. Cells were infected with a multiplicity of infection (MOI) of 2 with seasonal H3N2 virus, pH1N1 virus or HPAIV H5N1 for 1 hour. After 8 hours post infection (hpi), cells were fixed in the plate with 80% acetone and stored at −20°C. Plates were thawed, and washed with PBS before incubation with the primary antibody, a mouse-anti-influenza A nucleoprotein (Hb65) followed by peroxidase-labeled goat-anti-mouse IgG2a. Peroxidase was revealed using AEC resulting in a red precipitate. Influenza virus positive and -negative cells were counted in five 40x high power fields.

### Virus production

AM (n = 6), MM (n = 6) and GM-MM (n = 4) were seeded in a concentration of 100 000 cells per well and infected with a MOI of 0.1 with seasonal H3N2 virus, pH1N1 virus or HPAIV H5N1 for 1 hour. Supernatants were sampled at 4, 8 and 24 hpi. Virus titrations on these supernatants were performed by end-point titration in MDCK cells as described previously [36].

### Cytokine production after virus inoculation or LPS exposure

AM (n = 6 per treatment) and MM (n = 6 per treatment) were seeded in a concentration of 100 000 cells per well and infected with an MOI of 2 with seasonal H3N2 virus, pH1N1 virus or HPAIV H5N1 for 1 hour, or exposed to 100 ng/ml LPS for 1 hour. At 8 and 24 hpi, cells were harvested in lysis buffer, and mRNA was isolated using an mRNA capture kit (Roche Diagnostics Netherlands, Almere, Netherlands) according to manufacturer's instructions. Subsequently, cDNA was made using Superscript III reverse transcriptase (Invitrogen). Taqman analysis was used to determine target gene RNA expression as described previously [37]. Gene expression levels were corrected for GAPDH mRNA and ubiquitin C (UBC) mRNA levels and PCR efficiency, and expressed in fold induction (FI) over uninfected cells sampled at the same time point. Sequences of the PCR primers (Eurogentec, Maastricht, The Netherlands) are: forward primer 5′-TCCACTGGCGTCTTCAC, reverse primer 5′-GGCAGAGATGATGACCCTTTT for GAPDH; forward primer 5′-GGCAAAGATCCAAGATAAGGAA, reverse primer 5′-GGACCAAGTGCAGAGTGGAC for UBC; forward primer 5′-GAGCCCAAGGGCTACCAT, reverse primer 5′-GGGTTATGCTGGTTGTACAGG for TGF-beta; forward primer 5′-CAGCCTCTTCTCCTTCCTGAT, reverse primer 5′-GCCAGAGGGCTGATTAGAGA for TNF-alpha. Probes from the universal probe library from Roche were used; probe 45 for GAPDH; probe 11 for UBC; probe 29 for TNF-alpha and TGF-beta.

### Statistics

Statistical analyses were performed using SPSS 15 software. The Wilcoxon test was used for analysis of paired samples, and the Mann-Whitney test for unpaired samples.

### Accession numbers

TNF-alpha – NM_000594.2 (NCBI); TGF-beta – NM_000660.3 (NCBI); GAPDH – NM_002046.3 (NCBI) and UBC – NM_021009.4 (NCBI).
